# The Severe Typhoid Fever in Africa Program Highlights the Need for Broad Deployment of Typhoid Conjugate Vaccines

**DOI:** 10.1093/cid/ciz637

**Published:** 2019-10-30

**Authors:** Megan E Carey, A Duncan Steele

**Affiliations:** 1 Enteric and Diarrheal Diseases, Global Health, Bill & Melinda Gates Foundation, Seattle, Washington; 2 Department of Medicine, University of Cambridge, Cambridge, United Kingdom

**Keywords:** typhoid fever, enteric fever, surveillance, typhoid conjugate vaccine, vaccine introduction

## Abstract

The Typhoid Surveillance in Africa Program (TSAP) and the Severe Typhoid Fever in Africa (SETA) program have refined our understanding of age and geographic distribution of typhoid fever and other invasive salmonelloses in Africa and will help inform future typhoid control strategies, namely, introduction of typhoid conjugate vaccines.

Until relatively recently, the burden of enteric fever caused by *Salmonella* in Africa was not well described. A 2004 review of global enteric fever epidemiology data showed that only 2 countries in Africa (South Africa and Egypt) conducted systematic population-based enteric fever surveillance between 1954 and 2000 [1, 2], the outputs of which provided the basis for the modeled incidence estimate of 50 cases per 100 000 for the entire African continent. Recognizing this lack of representative incidence data, the World Health Organization (WHO) called for the generation of additional epidemiological data from sub-Saharan Africa [3]. Subsequently, with funding from the Bill & Melinda Gates Foundation, the International Vaccine Institute initiated the Typhoid Surveillance in Africa Program (TSAP) in 2009, establishing population-based surveillance in ten countries (Burkina Faso, Ethiopia, Ghana, Guinea-Bissau, Kenya, Madagascar, Senegal, South Africa, Sudan, and Tanzania), spanning urban, rural, and periurban settings. Notably, TSAP showed that typhoid fever incidence varied widely across the African continent, although the observed overall incidence of typhoid fever in Africa was 2–3 times higher than previous estimates [4, 5]. Furthermore, high incidence of typhoid fever was observed in both urban and rural settings, and higher disease incidence was observed among children in rural areas of Ghana as compared to urban areas, illustrating the spatiotemporal nature of the disease [6]. In addition, almost half (47%) of all isolates exhibited resistance to first-line antimicrobials [5], further emphasizing the need for broad deployment of typhoid conjugate vaccine (TCVs) to accelerate typhoid control through the prevention of drug-resistant infections.

The Severe Typhoid Fever in Africa (SETA) study was initiated in 2016 to generate additional data on the clinical and economic burden of severe typhoid disease and associated sequelae and to describe antimicrobial resistance (AMR) patterns. SETA is currently enrolling in six countries (Burkina Faso, Ghana, Madagascar, Ethiopia, Democratic Republic of Congo [DRC], and Nigeria). Similar efforts are ongoing in Asia as part of the Surveillance for Enteric Fever in Asia Project (SEAP), another prospective, population-based surveillance study, which is generating incidence rates and cost-of-illness data, and characterizing the burden of sequelae associated with severe enteric fever in Bangladesh, Nepal, and Pakistan [7]. Recently, the Severe Enteric Fever in India (SEFI) project was launched to generate data on the burden of disease in India. All of these multisite studies combine facility-based surveillance with healthcare utilization surveys to generate population denominators for incidence rate calculations. This hybrid approach to generating population-level incidence rates is less resource-intensive than a cohort study, but requires the application of adjustment factors, including proportion of study population seeking care at study facilities, proportion of eligible subjects enrolled, and sensitivity of blood culture, all of which create some uncertainty around incidence estimates [8–11]. The Strategic Typhoid Alliance Across Africa and Asia (STRATAA) study is generating incidence estimates for disease caused by typhoidal salmonellae infections in Malawi, Bangladesh, and Nepal using a census-defined population denominator, as well as conducting serosurveys to identify chronic carriers and assess population seroprevalence [12].

Data from these and other recent studies from Asia and Africa informed the WHO’s Strategic Advisory Group of Experts’ recent recommendation for use of TCV for primary vaccination of infants and children from 6 months of age, with an option for catch-up campaigns in children up to 15 years of age in areas of high endemicity [13]. Gavi, the Vaccine Alliance opened a 5-year, $85 million funding window to support introduction of TCV into routine immunization schedules as well to cover costs associated with catch-up campaigns [14], and the first TCV was prequalified by the WHO in late 2017 [15].

The high disease burden observed, coupled with the availability of a WHO Prequalified TCV and Gavi support, mean that there is massive potential impact of TCV introduction. Important TCV performance data are being generated by the Typhoid Vaccine Acceleration Consortium (TyVAC), which is conducting large-scale efficacy studies in Nepal, Malawi, and Bangladesh [16]. Additional interventions using Bharat Biotech’s Typbar-TCV are ongoing in Navi Mumbai, India, where the municiple government requested vaccine intervention [17] and in Pakistan in response to the ongoing outbreak of extensively drug-resistant (XDR) *Salmonella enterica* serovar Typhi. Additional large-scale vaccine studies will launch in 2020 in two African countries, Ghana and the DRC, under the coordination of the University of Cambridge and International Vaccine Institute, with funding from the European and Developing Countries Clinical Trials Partnership and the Bill & Melinda Gates Foundation. Importantly, there is a strong pipeline of new TCV candidates, with three additional manufacturers conducting late-stage clinical trials, which will contribute to increased supply security and price competition [18].

## THE SEVERE TYPHOID FEVER SURVEILLANCE PROGRAM IN AFRICA

This supplement introduces the methodology of the ongoing SETA program, which was designed to focus on the impact of severe invasive *Salmonella* infections at a mixture of urban and rural sites in Burkina Faso, the DRC, Ethiopia, Ghana, Madagascar, and Nigeria. Using standardized inclusion criteria, patients with suspected typhoid fever are enrolled at study facilities, where blood and stool samples are collected to identify causal agents, characterize AMR, and detect acute carriage [19]. In addition, urine samples are collected to determine previous treatment with antimicrobial agents, and oropharyngeal swab samples are collected to assess group A *Streptococcus* carriage. In cases where suspected intestinal perforations require surgical intervention, blood and tissue samples are also being collected when possible for testing using culture, polymerase chain reaction, and/or histopathological and cytological methods. Enrolled subjects with blood culture–confirmed invasive *Salmonella* infection are followed for 1 year to answer questions about long-term sequelae, host immunity, and chronic carriage. Postmortem surveys are also being conducted in selected study sites to assess potential mortality attributable to invasive *Salmonella* infection. Healthcare utilization surveys are being conducted to estimate the proportion of the catchment population seeking care at study facilities, as well as to collect information on socioeconomic status and other potential risk factors. Data are also being collected on cost of illness in selected study sites that will ultimately support cost-effectiveness analyses for different TCV introduction strategies [20]. Having recent cost-of-illness data from African settings, particularly for complicated cases of typhoid fever, should help inform decision making as well.

The authors summarize key published data through three systematic reviews—one on published typhoid fever data from Africa since 1950, one on spatial and temporal patterns of typhoid and paratyphoid fever outbreaks globally between 1990 and 2016, and one on frequency of complications associated with severe disease. The first reports that 42 of 57 African countries have reported at least 1 typhoid fever case during this time period, with the total number of reports increasing over time [21]. The second unsurprisingly describes the high proportion (66%) of outbreaks that are causally linked to contaminated water [22]. Both articles demonstrate the high spatiotemporal variability of typhoid disease, which has significant implications for vaccination strategies and other control measures. The third meta-analysis showed that 25% of hospitalized typhoid fever patients had complications, and a 36% higher prevalence of complications among those patients reporting having symptoms for ≥10 days at time of hospitalization [23] All three studies are limited by lack of standardized, comparable surveillance data and systems.

The authors in this supplement also highlight some site-specific data from TSAP and SETA. Teferi et al describe the etiology of acute febrile illness in children <15 years of age at Butajira Hospital in south-central Ethiopia, where 13.5% of febrile pediatric patients were malaria positive, and only 1.5% had blood culture–confirmed typhoid fever [24]. The authors detail some of the challenges associated with establishing systematic blood culture surveillance in this setting, including high contamination rates (11.9%) and limited quality control systems. Im et al describe two consequent outbreaks of dengue fever in Ouagadougou in 2016 and 2017 detected at a SETA study laboratory [25]. Data from Ibadan, Nigeria, in 2017 show high rates of bacteremia (17.0%) in febrile patients presenting to healthcare facilities, with *Staphylococcus aureus* (59.5%) and *Salmonella enterica* (28.4%) as the most commonly isolated bacterial species [26]. These data illustrate the variability in etiology of acute febrile disease across different African settings. Jeon at al make the case that nationally representative typhoid incidence data should, when available, inform vaccination strategies, and also reference this spatiotemporal variability in typhoid disease burden as one of the major difficulties associated with risk-based approaches [27].

Importantly, Toy et al summarize the prevalence of extended-spectrum β-lactamase (ESBL)–producing gram-negative bacterial isolates from 12 TSAP sites collected between 2010 and 2014 [28]. Analysis of 505 isolates showed that 12.1% demonstrated phenotypic ESBL activity, which was more prevalent in infants than in the broader population. Of note, carbapenems were shown to be the only remaining treatment option for some Enterobacteriaceae, which are often in short supply, expensive, and require parenteral administration. These data underscore the importance of AMR surveillance, which should inform local antimicrobial prescription behavior, as well as the urgent need for preventative interventions like vaccines.

## CURRENT AND FUTURE IMPLICATIONS OF SETA

With additional burden data, the availability of vaccines, and supportive funding in hand, the global community is uniquely positioned to achieve extraordinary progress toward short-term typhoid control. Ideally, sustained investment in water and sanitation infrastructure and maintenance would also be incorporated to amplify the effect of TCVs on typhoid control. Still, important questions remain for African countries considering introduction of TCVs on typhoid control, particularly those countries where even the minimal passive, sentinel, blood culture surveillance has not been established. These countries must rely on other sources of data, including but not limited to modeled data, presence of known risk factors (eg, lack of access to safe water and improved sanitation), or the presence of laboratory-confirmed typhoid in neighboring countries.

In addition to furnishing important data to countries included in the studies and providing additional data points to strengthen modeled estimates, multisite surveillance studies such as SETA provide an important platform to validate new, low-cost surveillance methods, including environmental surveillance and serological markers of infection, against the current gold standard of blood culture surveillance. Assessment of such methods in the context of established blood culture surveillance can yield important data about sensitivity, specificity, and even acceptability of these new surveillance methods for decision making. Such methods will likely be important to countries that are unable to establish sentinel blood culture surveillance but are still interested in assessing local incidence of enteric fever in service of decision making around TCV use.

Even with convincing burden data, decision-makers in country still must engage in a tradeoff exercise when considering introducing a new vaccine. Vaccine financing, particularly for Gavi graduating countries, and the strain that a new vaccine introduction can place on an already stressed immunization system must be weighed against the economic and health impact of a new program. Additional information about prevalence of severe disease and AMR, cost of illness, and mortality data are critically important to providing a fuller picture of the value of a vaccination program. SETA is generating such data on enteric fever in Africa, including better estimates of typhoid fever–associated mortality [19], which will be critical to updating cost-effectiveness analyses and supporting informed decision making around TCV introduction in Africa.

Leveraging platforms established by studies like SETA and SEAP to conduct ongoing AMR surveillance is of vital importance to understanding global disease epidemiology and informing national decision making. Established blood culture surveillance conducted by Aga Khan University, Pakistan facilitated the rapid detection of the spread of the XDR outbreak of *S.* Typhi from Hyderabad to Karachi. SEAP is also generating cost-of-illness estimates for the XDR typhoid strain in Pakistan, which has resulted in significantly longer hospital stays and can only be treated by one readily available oral antimicrobial [15]. Identification of such resistant strains, either in-country or in a neighboring country, would justifiably increase the urgency of TCV introduction. SETA is incorporating similar analyses in Africa.

The SETA sites will also provide a critical platform to demonstrate the value of the immunization program for ministries of health and Gavi, through TCV impact assessments and other evaluations. Established blood culture surveillance and associated laboratory infrastructure, trained personnel, and a well-defined catchment area will facilitate the conduct of future TCV impact assessments. This will also provide an important opportunity for countries to evaluate different vaccination strategies, looking at the impact of routine immunization plus catch-up campaigns in different areas of countries, or targeting different age groups.

Finally, SETA is generating important information about the burden of other invasive salmonelloses in Africa. TSAP showed significant numbers of invasive nontyphoidal *Salmonella* (iNTS) cases [5], and iNTS disease continues to constitute a high proportion of invasive bacterial infections in Africa. SEAP and other studies will also provide important data about the burden of enteric fever attributable to *Salmonella enterica* serovar Paratyphi A in Asian sites. These data will help inform future directions for broadly protective *Salmonella* vaccine prioritization and development.

## CONCLUSIONS

Systematic surveillance studies such as TSAP/SETA, SEAP, and others have done more than just broaden the global community’s understanding of age and geographic distribution of enteric fever. Preliminary data from such studies have informed key global policy decisions and will continue to guide optimal TCV use and future broadly protective *Salmonella* vaccine development efforts. Now, it is the responsibility of national governments and their advisory bodies to prioritize the implementation of TCVs for short-term control of the disease and reduction of transmission of the pathogen in vulnerable communities. These established surveillance sites will provide platforms for demonstrating the health impact of TCV programs.

## Notes

***Financial support*.** This research was funded by the Bill & Melinda Gates Foundation [OPP1127988]. The International Vaccine Institute acknowledges its donors including the Republic of Korea and the Swedish International Development Cooperation Agency. This publication was made possible through a grant from the Bill & Melinda Gates Foundation [OPP1201031].

***Supplement sponsorship*.** This article was published as part of the supplement “Severe Typhoid Fever in Africa (SETA) Program” sponsored by the International Vaccine Institute.

***Potential conflicts of interest.*** The authors report no potential conflicts of interest. Both authors have submitted the ICMJE Form for Disclosure of Potential Conflicts of Interest. Conflicts that the editors consider relevant to the content of the manuscript have been disclosed.
